# Characterization of Modal Frequencies and Orientation of Axisymmetric Resonators in Coriolis Vibratory Gyroscopes

**DOI:** 10.3390/mi12101206

**Published:** 2021-10-01

**Authors:** Xukai Ding, Han Zhang, Libin Huang, Liye Zhao, Hongsheng Li

**Affiliations:** 1 School of Instrument Science and Engineering, Southeast University, Nanjing 210096, China; huanglibin@seu.edu.cn (L.H.); liyezhao@seu.edu.cn (L.Z.); hsli@seu.edu.cn (H.L.); 2 Key Laboratory of Micro-Inertial Instruments and Advanced Navigation Technology, Ministry of Education, Nanjing 210096, China; 3 Institute of Electronic Engineering and Optoelectronic Technology, Nanjing University of Science and Technology Zijin College, Nanjing 210023, China; zhanghanqjxf@163.com

**Keywords:** frequency sweep, nonlinear coefficient, modal frequencies, MEMS gyroscope, modal orientation, ring down

## Abstract

This paper presents the characterization of the modal frequencies and the modal orientation of the axisymmetric resonators in Coriolis vibratory gyroscopes based on the approaches of the frequency sweep and the ring down. The modal frequencies and the orientation of the stiffness axis are the key parameters for the mechanical correction of the stiffness imperfections. The frequency sweep method utilizes the zero and the poles in the magnitude-frequency responses of the two-dimensional transfer function to extract the modal orientation information within the frequency domain. The ring down method makes use of the peak and the valley values of the beat signals at the readout electrodes to obtain the modal orientation and the coefficient of the nonlinear stiffness directly within the time domain. The proposed approaches were verified via a silicon ring resonator designed for gyroscopic sensing and the modal information from the experiments exhibited a good agreement between the methods of the frequency sweep and the ring down.

## 1. Introduction

Microelectromechanical system (MEMS) Coriolis vibratory gyroscopes (CVGs) have been revealed enormous potential for high-end applications, even for the applications of near-navigation grade [1,2,3,4,5]. Among the MEMS gyroscopes with the top performances, the MEMS CVGs with axisymmetric resonators, for instance, ring [6] or disk [7,8,9], hemispherical [10,11,12], and quad-mass [13,14,15] resonators, are particularly appealing by virtue of their angular rate-integrating ability, which offers benefits of high dynamics, wide ranges, and shock and vibration resistance. Besides, the symmetry in the structure of the resonators provides possibilities of various in-run calibration and compensation algorithms in the control system [16,17,18,19], further improving the bias and the scale factor performances for the gyroscopes.

The most significant property of the axisymmetric resonators is the symmetry between the two working modes of the gyroscopic operation. The efficiency of the energy transfer between the working modes primarily depends on the modal symmetry. However, the errors and imperfections in the fabrication and assembly processes are inevitable and will introduce the modal frequency split. It is essential to correct these errors after the fabrication for improving the gyroscope performances. Generally, the post-fabricated correction can be performed electrostatically or mechanically. Commonly, the electrostatic correct is actively and temporarily applied during the gyroscopic operation. Nonetheless, the mechanical correction is performed before the resonator is packaged and is permanent. Researchers have developed numerous approaches, such as mass deposition [20,21,22], laser ablation [23,24,25], ion beam trimming [14] and chemical etching [26], to mechanically correct the modal errors to diminish the frequency split. To accurately perform the mechanical correction, it is vital to find the precise position, namely, the modal orientation, where the deposition, the ablation, the trimming, or the etching should be carried out.

However, as far as we know, very few literatures particularly address the characterization of the modal orientation of the axisymmetric resonators. Oriented to the Jet Propulsion Laboratory MEMS vibratory rate gyroscope, the researchers reported an experimental system identification method to estimate the orientation of the stiffness matrix principal axes [27]. The proposed procedure includes first applying modern system identification algorithms to determine the multivariable input-output model and then the orientation of the principal axes was determined based on the identified model. Different from the methods in [27], this paper provides detailed approaches to characterize the modal frequencies and the modal orientation based upon the frequency sweep and upon the ring down method, which can be respectively performed in the frequency domain and the time domain, without any sophisticated modern system identification algorithms. The proposed approaches provide the necessary information utilized in the mechanical trimming processing.

The coming sections of this paper are organized as follows. Section 2 first reviews the dynamics of the axisymmetric resonators with respective to the displacements along the orthogonal working modes and those along the directions of capacitive forcing and pick-off, respectively. The second part of Section 2 introduces the two-dimensional transfer functions, in terms of the forcing frequency and the forcing direction, of the axisymmetric resonators. Section 3 reveals the relationships between the modal frequencies, the modal orientation, and the poles and the zero in the two-dimensional transfer function described in Section 2. Through the obtained relationships, the method based on the continuous linear frequency sweep to extract the modal characters is analyzed in detail. Section 4 gives the characterization approach based on the ring down method, which may be more effective as for resonators with high Q values. In addition, the ring down method can extract not only the modal frequencies and the orientation but also the nonlinear coefficients of the stiffness. Section 5 demonstrates the experimental verification of the methods discussed in Section 3 and Section 4 by means of a ring resonator. Finally, Section 6 briefly concludes this paper.

## 2. Dynamics and 2D Transfer Function of Axisymmetric Resonators in CVGs

### 2.1. Equations of Motion of Axisymmetric Resonators’ Working Modes

**Figure 1:**
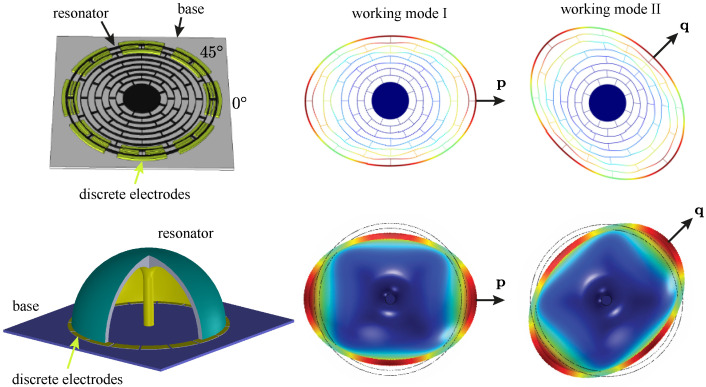
Mode shape with the wavelength of n=2 in a micro multi-ring resonator and a micro hemispherical resonator.

The mode shape with the wave number of n=2, shown in Figure 1 where a micro ring resonator and a micro hemispherical resonator are taken as examples, is commonly implemented as working modes in MEMS axisymmetric gyroscopes. From Figure 1, we can observe that there are two orthogonal modes for the mode shape of n=2. The angle between the principal axes of the working modes is $45^{\circ }$ in geometry, along the directions of p and q.

The fundamental equations of motion of the working modes can be described as two coupled oscillators

(1) $$\left[\begin{array}{c} \ddot{p}\left(t\right)\\ \ddot{q}\left(t\right) \end{array}\right]+2k\Omega \left[\begin{array}{cc} 0 & -1\\ 1 & 0 \end{array}\right]\left[\begin{array}{c} \dot{p}\left(t\right)\\ \dot{q}\left(t\right) \end{array}\right]+\left[\begin{array}{cc} \omega_{1}^{2} & 0\\ 0 & \omega_{2}^{2} \end{array}\right]\left[\begin{array}{c} p(t)\\ q(t) \end{array}\right]=0,$$

where p(t) and q(t) are displacements of the oscillator along p and q, *k* is an angular gain factor determined by the geometry of the oscillator, Ω is the applied angular rate, $\omega_{1}$ and $\omega_{2}$ are the modal frequencies of the working modes. The external forces and the damping forces are not presented in Equation (1). For convenience, we assign $\omega_{1}\leq \omega_{2}$ in this paper.

According to different control approaches for the working modes, CVGs with axisymmetric resonators can operate under the force-to-rebalance (FTR) scheme or the whole angle (WA) scheme. Under the FTR scheme, one of the working modes (called the primary mode) will be driven into resonance with a constant vibration amplitude. Once the gyroscope is subject to any rotation, the energy will transfer from the primary mode to the other (the secondary mode), making the latter vibrate as well. The control loop picks off the vibration induced by the Coriolis effect and forces the secondary mode stationary again. The force utilized to rebalance the secondary mode is taken as the measurement of the angular rate information.

Under the WA scheme, both of the working modes participate in in-phase oscillations, forming a standing wave whose azimuth will proportionally rotate with the input rotation. Therefore, the azimuth of the standing wave can be a measure of the angle rotated.

**Figure 2:**
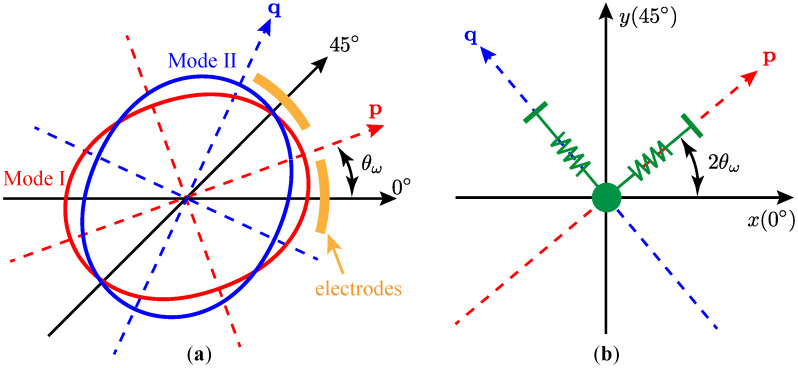
Displacements of the resonator. (**a**) in geometry; (**b**) from the perspective of capacitive readout.

For both the FTR scheme and the WA scheme, the resonator is expected perfectly axisymmetry, presenting the mode-matching condition, namely, $\omega_{1}=\omega_{2}$. However, as a result of structure imperfections introduced by fabrication errors and material defects, the parameters between the two working modes slightly differ from each. The imperfections will cause the azimuth of the working modes’ principal axes towards a specific direction, $\theta_{\omega }$, which is regularly misaligned with the center of the discrete electrodes, as illustrated in Figure 2a.

In this paper, $\theta_{\omega }$ represents the orientation of the principal axis with the lower frequency, namely $\omega_{1}$, and is defined within the range from −π/4 to π/4.

Although the motions of the resonator can be simply decomposed into the vibrations along the principal axes, only the displacements along the pick-off electrodes can be readout effectively. Figure 2b shows the relationship between the displacements along the principal axes (p and q) and those along the electrode axes, $0^{\circ }$ (*x*) and $45^{\circ }$ (*y*). It should be noted that the azimuth of the principal axis in the Cartesian coordinate is twice of that in the geometry, due to the mapping of the coordinate *y* from $45^{\circ }$ to $90^{\circ }$. Therefore, we can readily have

(2) $$\left[\begin{array}{c} p(t)\\ q(t) \end{array}\right]=R_{\omega }\left[\begin{array}{c} x(t)\\ y(t) \end{array}\right],$$

where x(t) and y(t) are the displacements along $0^{\circ }$ and $45^{\circ }$, and $R_{\omega }$ is a rotation matrix defined as

(3) $$R_{\omega }=\left[\begin{array}{cc} \cos2\theta_{\omega } & \sin2\theta_{\omega }\\ -\sin2\theta_{\omega } & \cos2\theta_{\omega } \end{array}\right].$$

By combining Equation (1) and Equation (2), from the perspective of the capacitive readout and drive, we can write the equations of motion as

(4) $$\left[\begin{array}{c} \ddot{x}\left(t\right)\\ \ddot{y}\left(t\right) \end{array}\right]+R_{\omega }^{T}\left[\begin{array}{cc} \omega_{1}^{2} & 0\\ 0 & \omega_{2}^{2} \end{array}\right]R_{\omega }\left[\begin{array}{c} x(t)\\ y(t) \end{array}\right]=0,$$

where Ω is omitted for the purpose of parameter characterizations in this paper.

If we further include the damping forces and the external electrostatic forces, the dynamics of the resonator can be described as

(5) $$\left[\begin{array}{c} \ddot{x}\left(t\right)\\ \ddot{y}\left(t\right) \end{array}\right]+R_{\tau }^{T}\left[\begin{array}{cc} 2/\tau_{1} & 0\\ 0 & 2/\tau_{2} \end{array}\right]R_{\tau }\left[\begin{array}{c} \dot{x}\left(t\right)\\ \dot{y}\left(t\right) \end{array}\right]+\left(R_{\omega }^{T}\left[\begin{array}{cc} \omega_{1}^{2} & 0\\ 0 & \omega_{2}^{2} \end{array}\right]R_{\omega }-\frac{K_{T}}{m}\right)\left[\begin{array}{c} x(t)\\ y(t) \end{array}\right]=\frac{1}{m}\left[\begin{array}{c} f_{x}\left(t\right)\\ f_{y}\left(t\right) \end{array}\right],$$

where $\tau_{1}$ and $\tau_{2}$ are time constants along the damping axes, $K_{T}$ is the stiffness matrix introduced by the electrostatic forces, *m* is the effective mass of the oscillator, $f_{x}\left(t\right)$ and $f_{y}\left(t\right)$ are electrostatic forces along the *x* and the *y* electrodes, respectively. In Equation (5), $R_{\tau }$ is a rotation matrix with respect to the azimuth of the damping axes and is in the form of

(6) $$R_{\tau }=\left[\begin{array}{cc} \cos2\theta_{\tau } & \sin2\theta_{\tau }\\ -\sin2\theta_{\tau } & \cos2\theta_{\tau } \end{array}\right],$$

where $\theta_{\tau }$ is the azimuth of the damping axes. The electrostatic stiffness matrix can be represented as [28]

(7) $$K_{T}\approx \eta_{k}\left[\begin{array}{cc} V_{x,\;\mathrm{rms}}^{2} & 0\\ 0 & V_{y,\;\mathrm{rms}}^{2} \end{array}\right],$$

where $\eta_{k}$, in N/m/V${}^{2}$, is an efficiency coefficient from voltage to stiffness, $V_{x,\;\mathrm{rms}}$ and $V_{y,\;\mathrm{rms}}$ are the voltages in RMS applied on the *x* and the *y* electrodes. The electrostatic forces exerted on the electrodes are in the form of

(8) $$\left[\begin{array}{c} f_{x}\left(t\right)\\ f_{y}\left(t\right) \end{array}\right]=\eta_{f}\left[\begin{array}{c} V_{x}^{2}\left(t\right)\\ V_{y}^{2}\left(t\right) \end{array}\right],$$

where $\eta_{f}$, in N/V${}^{2}$, is an efficiency coefficient from voltage to force, $V_{x}\left(t\right)$ and $V_{y}\left(t\right)$ are the voltages on the electrodes.

Equation (5) gives the basic dynamics of the working modes of the axisymmetric resonators in MEMS CVGs, from which the characterizations of the modal parameters can start.

### 2.2. Two-Dimensional Transfer Function

Equation (5) describes the axisymmetric resonator’s equations of motion along the electrodes of capacitive forcing and pick-off. However, in some situations, especially when the gyroscope operates under the WA scheme, the dynamics along particular forcing directions are of more interest. Under the WA scheme, the control system of the CVG picks off the azimuth of the standing wave and exerts the electrostatic force just towards the direction of the motion to maintain a stable vibration. Meanwhile, the control system also suppresses any orthogonal vibration which undermines the standing wave. To better understand the motions of the axisymmetric resonator vibrating in different directions, we can examine the dynamics in Equation (5) through different forcing angles.

By utilizing the orthogonality of $f_{x}$ and $f_{y}$, we can reconstruct the electrostatic forces as

(9) $$\left[\begin{array}{c} f_{\theta }\left(t\right)\\ f_{\theta +\pi /4}\left(t\right) \end{array}\right]=R\left(\theta \right)\left[\begin{array}{c} f_{x}\left(t\right)\\ f_{y}\left(t\right) \end{array}\right],$$

where $f_{\theta }$ and $f_{\theta +\pi /4}$ are the electrostatic forces towards the forcing angles of θ and θ+π/4, and

(10) $$R\left(\theta \right)=\left[\begin{array}{cc} \cos2\theta & \sin2\theta \\ -\sin2\theta & \cos2\theta \end{array}\right].$$

Similarly, the displacements along the directions of θ and θ+π/4 relate to *x* and *y* through

(11) $$\left[\begin{array}{c} x(t)\\ y(t) \end{array}\right]=R{\left(\theta \right)}^{T}\left[\begin{array}{c} r_{\theta }\left(t\right)\\ r_{\theta +\pi /4}\left(t\right) \end{array}\right].$$

By substituting Equation (9) and Equation (11) into Equation (5), we will arrive at

(12) $$\begin{array}{c} \left[\begin{array}{c} {\ddot{r}}_{\theta }\left(t\right)\\ {\ddot{r}}_{\theta +\pi /4}\left(t\right) \end{array}\right]+R\left(\theta \right)R_{\tau }^{T}\left[\begin{array}{cc} 2/\tau_{1} & 0\\ 0 & 2/\tau_{2} \end{array}\right]R_{\tau }R{\left(\theta \right)}^{T}\left[\begin{array}{c} {\dot{r}}_{\theta }\left(t\right)\\ {\dot{r}}_{\theta +\pi /4}\left(t\right) \end{array}\right]\\ +R\left(\theta \right)\left(R_{\omega }^{T}\left[\begin{array}{cc} \omega_{1}^{2} & 0\\ 0 & \omega_{2}^{2} \end{array}\right]R_{\omega }-\frac{K_{T}}{m}\right)R{\left(\theta \right)}^{T}\left[\begin{array}{c} r_{\theta }\left(t\right)\\ r_{\theta +\pi /4}\left(t\right) \end{array}\right]=\frac{1}{m}\left[\begin{array}{c} f_{\theta }\left(t\right)\\ f_{\theta +\pi /4}\left(t\right) \end{array}\right]. \end{array}$$

Applying the Laplace transform to the both sides of Equation (12) yields

(13) $$\left[\begin{array}{c} R_{\theta }\left(s\right)\\ R_{\theta +\pi /4}\left(s\right) \end{array}\right]=\left[\begin{array}{cc} T_{11}\left(s,\theta \right) & T_{12}\left(s,\theta \right)\\ T_{21}\left(s,\theta \right) & T_{22}\left(s,\theta \right) \end{array}\right]\left[\begin{array}{c} F_{\theta }\left(s\right)\\ F_{\theta +\pi /4}\left(s\right) \end{array}\right],$$

where $R_{\theta }\left(s\right)$, $R_{\theta +\pi /4}\left(s\right)$, $F_{\theta }\left(s\right)$, and $F_{\theta +\pi /4}\left(s\right)$ respectively are the Laplace transforms of $r_{\theta }\left(t\right)$, $r_{\theta +\pi /4}\left(t\right)$, $f_{\theta }\left(t\right)$, and $f_{\theta +\pi /4}\left(t\right)$, and

(14) $$\begin{array}{c} \left[\begin{array}{cc} T_{11}\left(s,\theta \right) & T_{12}\left(s,\theta \right)\\ T_{21}\left(s,\theta \right) & T_{22}\left(s,\theta \right) \end{array}\right]=\frac{1}{m}\{s^{2}I_{2\times 2}+sR\left(\theta \right)R_{\tau }^{T}\left[\begin{array}{cc} 2/\tau_{1} & 0\\ 0 & 2/\tau_{2} \end{array}\right]R_{\tau }R{\left(\theta \right)}^{T}\\ +R\left(\theta \right)\left(R_{\omega }^{T}\left[\begin{array}{cc} \omega_{1}^{2} & 0\\ 0 & \omega_{2}^{2} \end{array}\right]R_{\omega }-\frac{K_{T}}{m}\right)R{\left(\theta \right)}^{T}{\}}^{-1}. \end{array}$$

By setting $F_{\theta +\pi /4}\left(s\right)=0$ in Equation (13), we can get

(15) $$\begin{array}{cc} R_{\theta }\left(s\right) & =T_{11}\left(s,\theta \right)\;F_{\theta }\left(s\right),\\ R_{\theta +\pi /4}\left(s\right) & =T_{21}\left(s,\theta \right)\;F_{\theta }\left(s\right), \end{array}$$

where $T_{11}\left(s,\theta \right)$ is the transfer function from the electrostatic force $f_{\theta }\left(t\right)$ to the displacement $r_{\theta }\left(t\right)$, and $T_{21}\left(s,\theta \right)$ actually reflects the cross-coupling from $f_{\theta }\left(t\right)$ to the displacement along θ+π/4. $T_{11}\left(s,\theta \right)$ and $T_{21}\left(s,\theta \right)$ can be obtained by matrix manipulations in Equation (14)

(16) $$\begin{array}{cc} T_{11}\left(s,\;\theta \right) & =\frac{1}{m}\frac{d(s,\;\theta )}{a(s,\;\theta )d(s,\;\theta )-b(s,\;\theta )c(s,\;\theta )}, \end{array}$$

(17) $$\begin{array}{cc} T_{21}\left(s,\;\theta \right) & =\frac{1}{m}\frac{-c(s,\;\theta )}{a(s,\;\theta )d(s,\;\theta )-b(s,\;\theta )c(s,\;\theta )}, \end{array}$$

where

(18) $$\begin{array}{c} a\left(s,\;\theta \right)=s^{2}+\left[\frac{2}{\tau_{1}}\cos^{2}2(\theta -\theta_{\tau })+\frac{2}{\tau_{2}}\sin^{2}2(\theta -\theta_{\tau })\right]s\\ +\omega_{1}^{2}\cos^{2}2(\theta -\theta_{\omega })+\omega_{2}^{2}\sin^{2}2(\theta -\theta_{\omega })-\frac{\eta_{k}}{m}\left(V_{x,\;\mathrm{rms}}^{2}\cos^{2}2\theta +V_{y,\;\mathrm{rms}}^{2}\sin^{2}2\theta \right), \end{array}$$

(19) $$\begin{array}{c} b\left(s,\;\theta \right)=c\left(s,\;\theta \right)=-\frac{1}{2}\left(\frac{2}{\tau_{1}}-\frac{2}{\tau_{2}}\right)\sin4(\theta -\theta_{\tau })s-\frac{\omega_{1}^{2}-\omega_{2}^{2}}{2}\sin4(\theta -\theta_{\omega })\\ +\frac{1}{2}\frac{\eta_{k}}{m}\left(V_{x,\;\mathrm{rms}}^{2}-V_{y,\;\mathrm{rms}}^{2}\right)\sin4\theta , \end{array}$$

and

(20) $$\begin{array}{c} d\left(s,\;\theta \right)=s^{2}+\left[\frac{2}{\tau_{1}}\sin^{2}2(\theta -\theta_{\tau })+\frac{2}{\tau_{2}}\cos^{2}2(\theta -\theta_{\tau })\right]s\\ +\omega_{1}^{2}\sin^{2}2(\theta -\theta_{\omega })+\omega_{2}^{2}\cos^{2}2(\theta -\theta_{\omega })-\frac{\eta_{k}}{m}\left(V_{x,\;\mathrm{rms}}^{2}\sin^{2}2\theta +V_{y,\;\mathrm{rms}}^{2}\cos^{2}2\theta \right). \end{array}$$

To gain some insights, if we ignore the damping mismatch and assume that $V_{x,\mathrm{rms}}=V_{y,\mathrm{rms}}=V_{\mathrm{rms}}$, then Equations (16) and (17) will be reduced to

(21) $$\begin{array}{cc} T_{11}\left(s,\;\theta \right) & \approx \frac{1}{m}\frac{s^{2}+2s/\tau_{0}+\omega_{1}^{2}\sin^{2}2\left(\theta -\theta_{\omega }\right)+\omega_{2}^{2}\cos^{2}2\left(\theta -\theta_{\omega }\right)-\frac{\eta_{k}}{m}V_{\mathrm{rms}}^{2}}{\left(s^{2}+2s/\tau_{0}+\omega_{1}^{2}-\frac{\eta_{k}}{m}V_{\mathrm{rms}}^{2}\right)\left(s^{2}+2s/\tau_{0}+\omega_{2}^{2}-\frac{\eta_{k}}{m}V_{\mathrm{rms}}^{2}\right)}, \end{array}$$

(22) $$\begin{array}{cc} T_{21}\left(s,\;\theta \right) & \approx \frac{1}{m}\frac{\frac{1}{2}\left(\omega_{1}^{2}-\omega_{2}^{2}\right)\sin4(\theta -\theta_{\omega })}{\left(s^{2}+2s/\tau_{0}+\omega_{1}^{2}-\frac{\eta_{k}}{m}V_{\mathrm{rms}}^{2}\right)\left(s^{2}+2s/\tau_{0}+\omega_{2}^{2}-\frac{\eta_{k}}{m}V_{\mathrm{rms}}^{2}\right)}, \end{array}$$

where $\tau_{0}$ is a time constant for the ideal damped decay.

**Figure 3:**
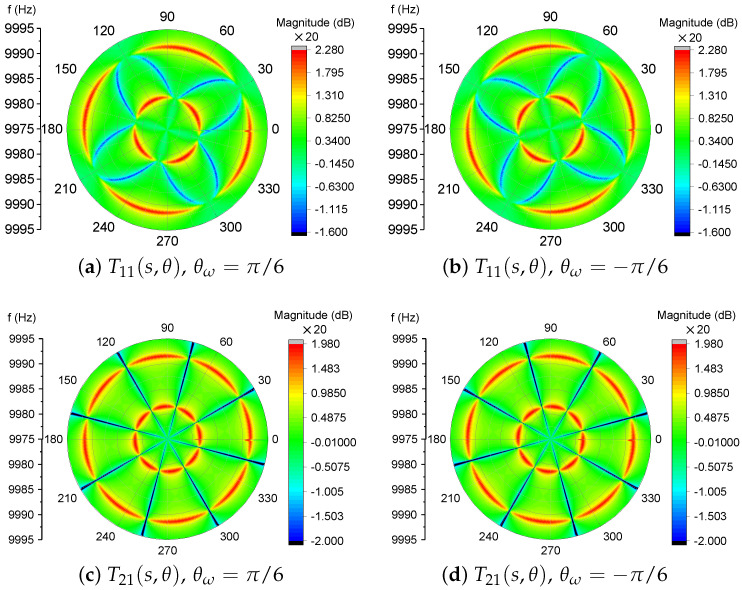
The magnitude-frequency responses of the 2D transfer function $T_{11}\left(s,\;\theta \right)$.

Figure 3 visualizes the magnitude-frequency responses of $T_{11}$ and $T_{21}$ as functions of both the forcing frequency, *f*, and the forcing angle, θ. The parameters used in Figure 3 are listed in Table 1.

Figure 3 clearly exhibits the changes of the magnitude responses over different forcing angles. In $T_{11}$, as shown in Figure 3a,b, the response will experience two peaks and one valley, except in the directions of θ+nπ/4 (0≤n≤7,n∈Z) where only one peak will occur at the frequency of $\omega_{1}$ or $\omega_{2}$. The responses of cross-couplings, in Figure 3c,d, display two resonances in most directions expect in θ+nπ/4 where no couplings occur at all, which can be verified by examining the numerator of Equation (22). Also, the most pronounced difference between $T_{11}$ and $T_{21}$ is the valley between the two peaks.

The peaks and the valley in the transfer function of $T_{11}$ can be utilized to reveal the modal frequencies and the orientation of the stiffness axes, which will be discussed in Section 3.

## 3. Modal Frequencies and Orientation of Stiffness Axes

### 3.1. Poles and Zeros in Magnitude-Frequency Response of the Transfer Function

It is clear to observe, in Figure 3, that the poles and the zero in the magnitude-frequency responses of the two-dimensional transfer function contain sufficient information about the modal frequencies and the orientation of the stiffness axes.

If the resonator is driven towards the electrodes at $0^{\circ }$, the transfer function of $T_{11}\left(s,0\right)$ can be obtained as

(23) $$T_{11}\left(s,\;0\right)=\frac{1}{m}\frac{s^{2}+2s/\tau_{0}+\omega_{1}^{2}\sin^{2}2\theta_{\omega }+\omega_{2}^{2}\cos^{2}2\theta_{\omega }-\frac{\eta_{k}}{m}V_{\mathrm{rms}}^{2}}{\left(s^{2}+2s/\tau_{0}+\omega_{1}^{2}-\frac{\eta_{k}}{m}V_{\mathrm{rms}}^{2}\right)\left(s^{2}+2s/\tau_{0}+\omega_{2}^{2}-\frac{\eta_{k}}{m}V_{\mathrm{rms}}^{2}\right)}.$$

**Figure 4:**
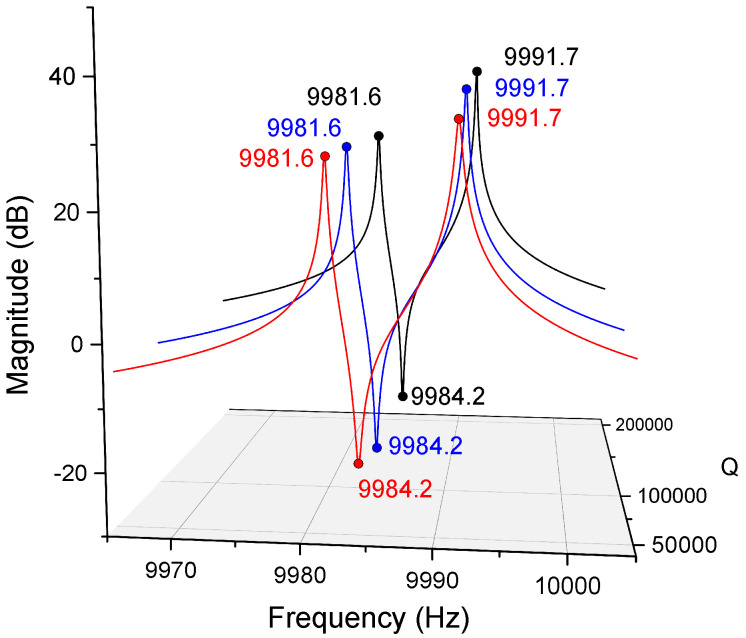
Magnitude-frequency responses of $T_{11}\left(s,0\right)$ with different damping mismatches.

In Equation (23), the errors in damping forces are still ignored because the damping mismatch will not significantly affect the modal frequencies and their directions. Figure 4 manifests the transfer function of $T_{11}\left(s,0\right)$ with different damping mismatches. The Q-factor of the working mode with the frequency of $\omega_{2}$ is fixed as 100,000. The corresponding poles and zeros of the magnitude-frequency responses almost stay at the same frequencies while the Q-factor of the frequency of $\omega_{1}$ changes from 50,000 to 200,000.

According to the fact demonstrated in Figure 4, regardless of the impacts of the damping errors on the modal frequencies, the locations of the poles and the zero in the transfer function can be found as

(24) $$\begin{array}{cc} \; & z^{2}=\omega_{1}^{2}\sin^{2}2\theta_{\omega }+\omega_{2}^{2}\cos^{2}2\theta_{\omega }-\frac{\eta_{k}}{m}V_{\mathrm{rms}}^{2},\\ \; & p_{1}^{2}=\omega_{1}^{2}-\frac{\eta_{k}}{m}V_{\mathrm{rms}}^{2},\\ \; & p_{2}^{2}=\omega_{2}^{2}-\frac{\eta_{k}}{m}V_{\mathrm{rms}}^{2}, \end{array}$$

from which we can attain the relation between the poles, the zero, and the modal orientation as

(25) $$\begin{array}{cc} z^{2} & =p_{1}^{2}\sin^{2}2\theta_{\omega }+p_{2}^{2}\cos^{2}2\theta_{\omega }\\ \; & =\frac{1}{2}\left(p_{1}^{2}+p_{2}^{2}\right)+\frac{1}{2}\left(p_{2}^{2}-p_{1}^{2}\right)\cos4\theta_{\omega }. \end{array}$$

However, by definition, the modal orientation $\theta_{\omega }$ is between −π/4 to π/4. Hence, Equation (25) only gives the absolute value of $\theta_{\omega }$

(26) $$\left|\theta_{\omega }\right|=\frac{1}{4}\arccos\left(\frac{2z^{2}-p_{1}^{2}-p_{2}^{2}}{p_{2}^{2}-p_{1}^{2}}\right).$$

To determine the direction of $\theta_{\omega }$, once the absolute value of $\theta_{\omega }$ is obtained, we can further drive the resonator towards $\theta_{\omega }$ and $-\theta_{\omega }$. The resultant magnitude-frequency response can be found as

(27) $$T_{11}\left(s,\;\theta_{\omega }\right)=\frac{1}{m}\frac{1}{s^{2}+2s/\tau_{0}+\omega_{1}^{2}-\frac{\eta_{k}}{m}V_{\mathrm{rms}}^{2}},$$

and

(28) $$T_{11}\left(s,\;-\theta_{\omega }\right)=\frac{1}{m}\frac{s^{2}+2s/\tau_{0}+\omega_{1}^{2}\sin^{2}4\theta_{\omega }+\omega_{2}^{2}\cos^{2}4\theta_{\omega }-\frac{\eta_{k}}{m}V_{\mathrm{rms}}^{2}}{\left(s^{2}+2s/\tau_{0}+\omega_{1}^{2}-\frac{\eta_{k}}{m}V_{\mathrm{rms}}^{2}\right)\left(s^{2}+2s/\tau_{0}+\omega_{2}^{2}-\frac{\eta_{k}}{m}V_{\mathrm{rms}}^{2}\right)}.$$

**Figure 5:**
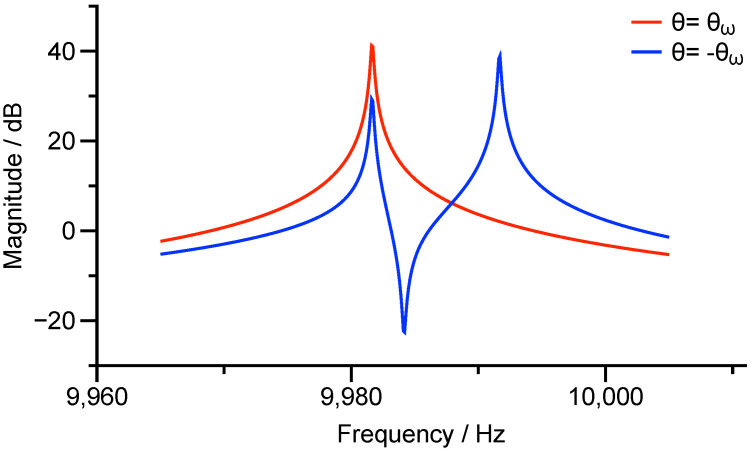
Magnitude-frequency responses of $T_{11}\left(s,\theta_{\omega }\right)$ and $T_{11}\left(s,-\theta_{\omega }\right)$.

There will be only one resonance in the response along the orientation of $\theta_{\omega }$ and will be still two resonances along $-\theta_{\omega }$, which is depicted in Figure 5.

### 3.2. Continuous Linear Frequency Sweep

Voltage with continuous linear frequency sweep, also named as the linear chirp signal, defined in Equation (29), can be performed to effectively extract the poles and the zero in the transfer function.

(29) $$V_{x}\left(t\right)=V_{d}+V_{a}\left(t\right),$$

where $V_{x}\left(t\right)$ is the voltage applied on the electrodes along $0^{\circ }$ direction, first introduced in Equation (8), $V_{d}$ is the direct current voltage, and

(30) $$V_{a}\left(t\right)=A\;\Pi \left(\frac{t}{T}-\frac{1}{2}\right)\cos\vartheta (t),$$

where *A* is the amplitude of the chirp signal, *T* is the duration time of the sweep, ϑ(t) is the instantaneous phase, Π(x) is the standard rectangle function and is defined as

(31) $$\Pi \left(x\right)=\left\{\begin{array}{c} 1,\;\mathrm{for}\;\;|x|<1/2,\\ 0,\;\mathrm{otherwise}. \end{array}\right.$$

**Figure 6:**
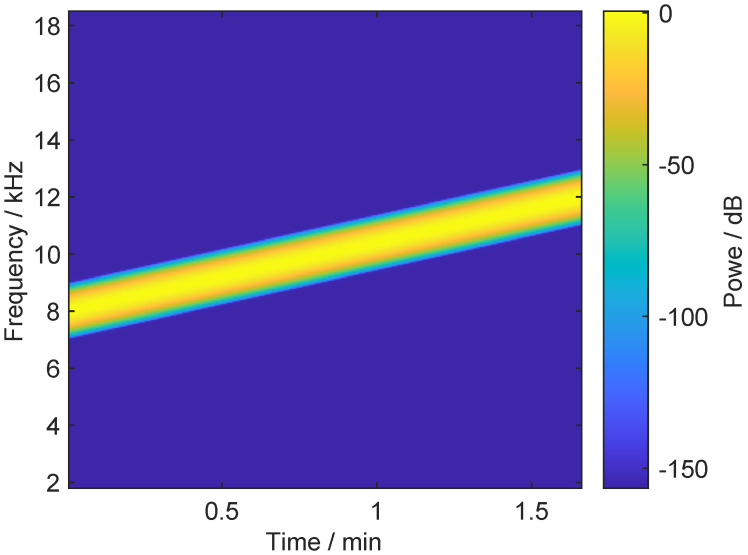
The time-frequency spectrogram of a chirp signal with parameters defined in Table 2.

The instantaneous angular frequency can be calculated as

(32) $$\begin{array}{cc} \omega (t) & =\frac{d}{dt}\vartheta \left(t\right)\\ \; & =\omega_{L}+\frac{\omega_{H}-\omega_{L}}{T}t,\;0\leq t\leq T \end{array}$$

where $\omega_{L}$ is the start angular frequency of the linear sweep, and $\omega_{H}$ is the end angular frequency. Figure 6 illustrates the chirp signal, with the parameters listed in Table 2, in the time-frequency domain. The instantaneous frequency of the signal linearly raises from 8 kHz to 12 kHz within 100 s.

By omitting the low amplitude oscillations and assuming that t≪T, we can approximate the autocorrelation function of Equation (30) as [29]

(33) $$C_{VV}\left(t\right)\approx \frac{A^{2}T}{2}\mathrm{sinc}\left(Wt\right)\cos\left(\omega_{c}t\right),$$

where *W* is the signal bandwidth in Hz, $\omega_{c}$ is the central angular frequency. We have the following definitions:

$$W=\left(\omega_{H}-\omega_{L}\right)/2\pi ,\;\omega_{c}=\left(\omega_{L}+\omega_{H}\right)/2,\;\mathrm{sinc}\left(x\right)=\sin\left(\pi x\right)/\pi x.$$

The Fourier transform of Equation (33) gives the energy spectral density of the chirp signal $V_{a}\left(t\right)$

(34) $$\Phi_{V_{a}}\left(\omega \right)=\frac{A^{2}T}{4W}\left[\Pi \left(\frac{\omega -\omega_{c}}{2\pi W}\right)+\Pi \left(\frac{\omega +\omega_{c}}{2\pi W}\right)\right],$$

where Π(x) is defined in Equation (31).

**Figure 7:**
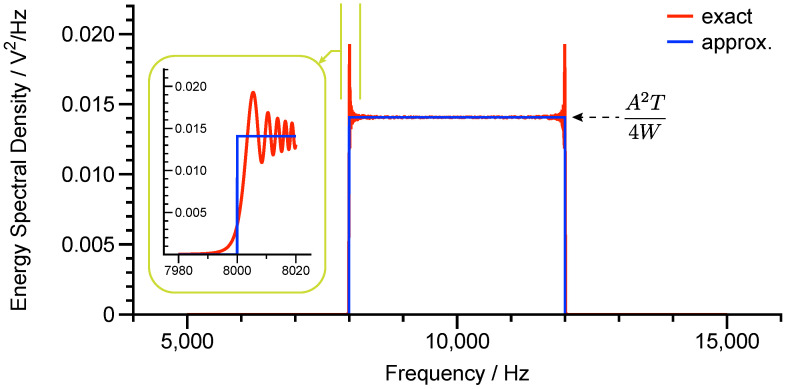
The energy spectral density of the chirp signal.

Figure 7 demonstrates the approximated energy spectral density of the chirp signal from Equation (34) and the exact curve from the numerical calculation. Except the oscillatory ripples near the edge frequencies, Equation (34) can retain the essential properties of the chirp signal’s energy spectral density.

To ensure the poles and the zero in the magnitude-frequency response can be covered, we set the relationship between the frequencies as

(35) $$\omega_{L}\ll \omega_{1}<\omega_{2}\ll \omega_{H}.$$

The amplitude spectral density of the displacement along $0^{\circ }$ direction can be acquired by

(36) $$\begin{array}{cc} \sqrt{\Phi_{x}\left(\omega \right)} & =2\eta_{f}V_{d}\sqrt{\Phi_{V_{a}}\left(\omega \right)}\;|T_{11}\left(j\omega ,0\right)|\\ \; & =\eta_{f}V_{d}A\sqrt{\frac{T}{W}}\;|T_{11}\left(j\omega ,0\right)|. \end{array}$$

Equation (36) indicates that, if ignoring the ripples at the edge frequencies, the amplitude spectral density of the chirp-driven resonator’s outcome just gives the curve of the frequency response. It should be noted that the strength of the resultant amplitude spectral density can be adjusted by the chirp input under the law of $A\sqrt{T/W}$. On the other hand, the resultant frequency resolution in $\Phi_{x}\left(j2\pi f\right)$ is inversely proportional to the sweep time *T*.

**Figure 8:**
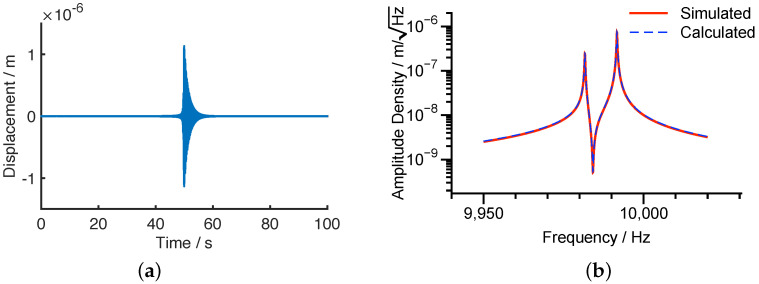
The responses of the resonator under the chirp excitation with A=0.1V, $V_{d}=5\;V$, $\eta_{f}=1\times 10^{-7}\;N/V^{2}$. (**a**) Time domain. (**b**) Frequency domain.

Figure 8 presents the simulated response to the chirp excitation in the time domain and in the frequency domain, respectively. The numerically simulated amplitude spectral density and that calculated from Equation (36) are also compared in Figure 8b, showing the full consistence.

Once the peaks and the valley in the transfer function of $T_{11}\left(s,0\right)$ are extracted through the chirp excitation along $0^{\circ }$, the absolute value of the modal orientation can be derived from Equation (26). The sign of the orientation can be conformed by the chirp excitation towards $\theta_{\omega }$ and $-\theta_{\omega }$, from which the magnitude responses of $T_{11}(s,\theta_{\omega }$) and $T_{11}(s,-\theta_{\omega })$ can be computed.

However, for resonators vibrating with considerable amplitudes, the stiffness force will exhibit the nonlinear property. The pronounced consequence of the nonlinearity on the frequency sweep is the hysteresis in the magnitude-frequency response, presenting sharp jumps at certain frequencies and, thus, making the positions of the poles indistinct in the frequency domain.

## 4. Ring-Down and Nonlinear Stiffness Coefficient

The modal frequencies, the orientation, and even the nonlinear stiffness coefficient, are hidden in the free oscillation signals of the resonator. First we introduce the undamped free-oscillation of the resonator along the p and q modes

(37) $$\begin{array}{cc} \; & P\left(t\right)=A_{1}\sin\left(\omega_{1}t\right),\\ \; & Q\left(t\right)=A_{2}\sin\left(\omega_{2}t+\phi \right), \end{array}$$

where $A_{1}$ and $A_{2}$ are the amplitudes of p and q modes’ oscillations, $A_{1,2}\geq 0$, and ϕ is the initial phase difference between them.

By combining Equation (37) and Equation (2), we get the undamped free-vibrations along the forcer electrodes and the readout electrodes as

(38) $$\begin{array}{cc} \; & X\left(t\right)=A_{1}\cos2\theta_{\omega }\sin\left(\omega_{1}t\right)-A_{2}\sin2\theta_{\omega }\sin\left(\omega_{2}t+\phi \right),\\ \; & Y\left(t\right)=A_{1}\sin2\theta_{\omega }\sin\left(\omega_{1}t\right)+A_{2}\cos2\theta_{\omega }\sin\left(\omega_{2}t+\phi \right). \end{array}$$

**Figure 9:**
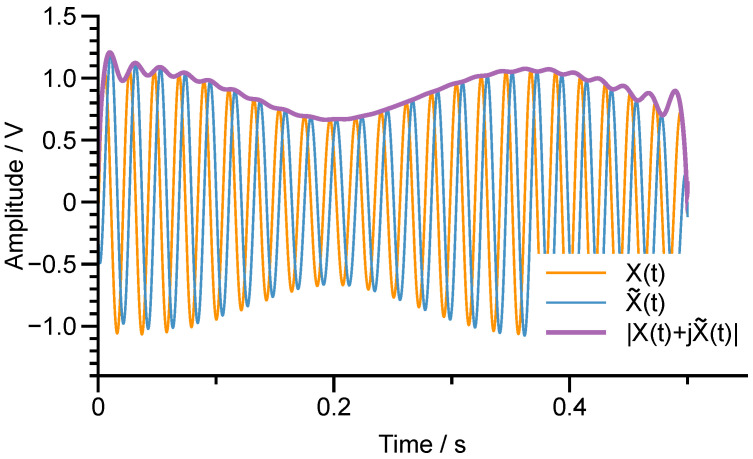
The beats in X(t) and the Hilbert transform of X(t).

There distinctly exhibits beats in X(t) and Y(t) since $\omega_{1}$ and $\omega_{2}$ are pretty close, as demonstrated in Figure 9.

To study the envelope of the beat signal, we can utilize the concept of the analytic signal

(39) $$X_{A}\left(t\right)=X\left(t\right)+j\;\tilde{X}\left(t\right),$$

where

(40) $$\tilde{X}\left(t\right)=H\left[X\left(t\right)\right]=\pi^{-1}\int_{-\infty }^{\infty }\frac{X(\tau )}{t-\tau }d\tau ,$$

which is defined as the Hilbert transform of X(t).

By the definition in Equation (40), the Hilbert transform of X(t) can be calculated as

(41) $$\tilde{X}\left(t\right)=A_{1}\cos2\theta_{\omega }\cos\left(\omega_{1}t\right)-A_{2}\sin2\theta_{\omega }\cos\left(\omega_{2}t+\phi \right).$$

Therefore, the envelope of X(t) can be attained by

(42) $$\begin{array}{cc} E_{X}\left(t\right) & =|\;X_{A}\left(t\right)\;|\\ \; & =\sqrt{X^{2}\left(t\right)+{\tilde{X}}^{2}\left(t\right)}\\ \; & =\sqrt{A_{1}^{2}\cos^{2}2\theta_{\omega }+A_{2}^{2}\sin^{2}2\theta_{\omega }-A_{1}A_{2}\sin4\theta_{\omega }\cos\left[\left(\omega_{2}-\omega_{1}\right)t+\phi \right]}. \end{array}$$

Figure 9 also shows the Hilbert transform of X(t) and the magnitude of the analytic signal $X_{A}\left(t\right)$, which exactly is the envelope of X(t). Equation (42) implies that the fundamental frequency of the signal envelope reflects the modal frequency split, $\omega_{2}-\omega_{1}$. Similarly, the envelope of Y(t) can be expressed by

(43) $$E_{Y}\left(t\right)=\sqrt{A_{1}^{2}\sin^{2}2\theta_{\omega }+A_{2}^{2}\cos^{2}2\theta_{\omega }+A_{1}A_{2}\sin4\theta_{\omega }\cos\left[\left(\omega_{2}-\omega_{1}\right)t+\phi \right]}.$$

**Figure 10:**
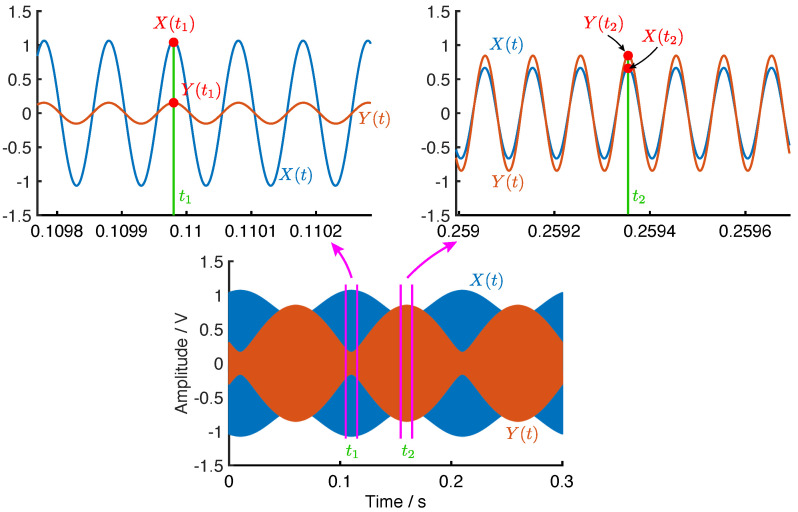
The beats in X(t) and Y(t).

By observing the beats in both X(t) and Y(t), demonstrated in Figure 10, we can find that, if $\theta_{\omega }>0$, when $t_{1}$ satisfies $\sin(\omega_{1}t_{1})=1$ and $\sin(\omega_{2}t_{1}+\phi )=-1$, $E_{X}\left(t\right)$ will reach the peak, that is

(44) $$A_{1}\cos2\theta_{\omega }+A_{2}\sin2\theta_{\omega }=X\left(t_{1}\right).$$

Meanwhile, at $t=t_{1}$ the value of Y(t) will be

(45) $$A_{1}\sin2\theta_{\omega }-A_{2}\cos2\theta_{\omega }=Y\left(t_{1}\right),$$

where $\left|Y(t_{1})\right|$ is indeed the minimum of $E_{Y}\left(t\right)$ but the sign of $Y(t_{1})$ should be determined by checking the instantaneous phase of Y(t).

On the other hand, when $t=t_{2}$ satisfies $\sin(\omega_{1}t_{2})=1$ and $\sin(\omega_{2}t_{2}+\phi )=1$, $E_{Y}\left(t\right)$ will reach the peak

(46) $$A_{1}\sin2\theta_{\omega }+A_{2}\cos2\theta_{\omega }=Y\left(t_{2}\right),$$

and X(t) is

(47) $$A_{1}\cos2\theta_{\omega }-A_{2}\sin2\theta_{\omega }=X\left(t_{2}\right).$$

**Figure 11:**
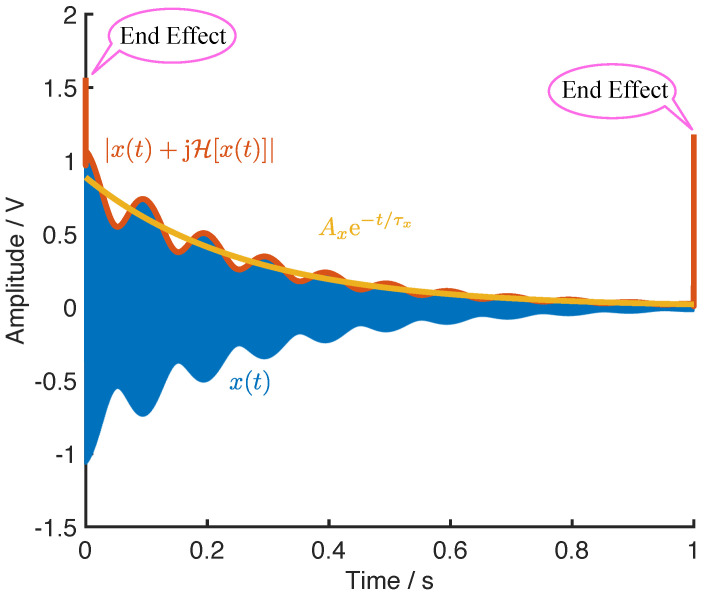
The ring-down beats and the envelope of the decayed beats.

The constraints described by Equations (44)–(47) are sufficient for resonators with extremely high Q-factor, whose time constant of the ring-down is large enough to perform the above analysis. However, if the ring-down of the resonators decays notably, the peak and the valley values of the beats are not easy to maintain, as shown in Figure 11.

The envelope of the ring-down beats, calculated from the magnitude of the analytic signal, are also depicted in Figure 11, where the exponential fitting curve of the envelope is also presented. It should be noted that the start point and the end point of the envelope exist serious errors due to the end effect of the Hilbert transform. Therefore, the fitting processing of the ring-down should exclude the start and the end of the envelope.

Based on the fitting curve we can obtain the time constant of the ring-down for both x(t) and y(t) as $\tau_{x}$ and $\tau_{y}$, respectively. Then, the undamped signals of X(t), Y(t), and their corresponding envelopes can be obtained by

(48) $$\begin{array}{cc} X\left(t\right)=x\left(t\right)\;e^{t/\tau_{x}}\;, & E_{X}\left(t\right)=E_{x}\left(t\right)\;e^{t/\tau_{x}}\;,\\ Y\left(t\right)=y\left(t\right)\;e^{t/\tau_{y}}\;, & E_{Y}\left(t\right)=E_{y}\left(t\right)\;e^{t/\tau_{y}}\;. \end{array}$$

According to the relations in Equation (48), the peak and the valley values of the beats can be acquired as the undamped cases. Once the essential values are determined effectively, we rewrite Equations (44)–(47) as

(49) $$\begin{array}{cc} \; & \varepsilon_{1}=A_{1}\cos2\theta_{\omega }+sign\left(\theta_{\omega }\right)A_{2}\sin2\theta_{\omega }-X\left(t_{1}\right),\\ \; & \varepsilon_{2}=A_{1}\sin2\theta_{\omega }-\mathrm{sign}\left(\theta_{\omega }\right)A_{2}\cos2\theta_{\omega }-Y\left(t_{1}\right),\\ \; & \varepsilon_{3}=\mathrm{sign}\left(\theta_{\omega }\right)A_{1}\cos2\theta_{\omega }-A_{2}\sin2\theta_{\omega }-X\left(t_{2}\right),\\ \; & \varepsilon_{4}=\mathrm{sign}\left(\theta_{\omega }\right)A_{1}\sin2\theta_{\omega }+A_{2}\cos2\theta_{\omega }-Y\left(t_{2}\right), \end{array}$$

where $\varepsilon_{1}$ to $\varepsilon_{4}$ can be interpreted as the residual errors, and the case for $\theta_{\omega }<0$ is also considered.

By minimizing the sum of the residual errors

(50) $$V\left(\theta_{\omega },A_{1},A_{2}\right)=\varepsilon_{1}^{2}+\varepsilon_{2}^{2}+\varepsilon_{3}^{2}+\varepsilon_{4}^{2},$$

we can find the estimation of the modal orientation as

(51) $${\hat{\theta }}_{\omega }=\theta_{1},\;\mathrm{or}\;{\hat{\theta }}_{\omega }=\theta_{1}-\mathrm{sign}\left(\theta_{1}\right)\frac{\pi }{4},$$

where $\theta_{1}$ satisfies $V\left(\theta_{1},A_{1},A_{2}\right)=\mathrm{MIN}\left\{V\left(\theta_{\omega },A_{1},A_{2}\right)\right\}$.

**Figure 12:**
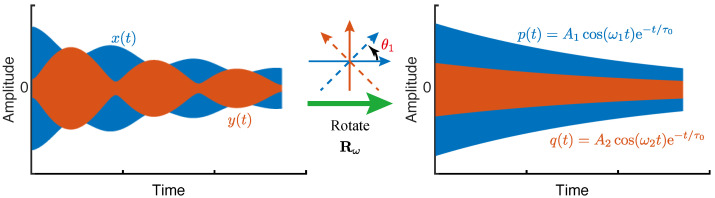
The vibrations along the principal axes rotated from the xy axes. If the obtained $\omega_{1}$ is less than $\omega_{2}$, then $\theta_{\omega }=\theta_{1}$; otherwise, $\theta_{\omega }=\theta_{1}-\mathrm{sign}\left(\theta_{1}\right)\pi /4$.

Similar to the method of the frequency sweep, the value of θ still cannot be figured out by one single constraint. However, by virtue of the obtained $\theta_{1}$, we can rotate the ring-down vibrations along xy to those along pq through Equation (2), as illustrated in Figure 12.

After the rotation, the signals along p and q will reduce back to $A_{1}\cos\left({\hat{\omega }}_{1}t\right)\exp\left(-t/\tau_{0}\right)$ and $A_{2}\cos\left({\hat{\omega }}_{2}t\right)\exp\left(-t/\tau_{0}\right)$. In addition, we can find that if a rotation of $\theta_{1}-\mathrm{sign}\left(\theta_{1}\right)\pi /4$ is performed, the signals along p and q would be $A_{2}\cos\left({\hat{\omega }}_{2}t\right)\exp\left(-t/\tau_{0}\right)$ and $A_{1}\cos\left({\hat{\omega }}_{1}t\right)$$\exp(-t/\tau_{0})$, instead. Nonetheless, we have defined $\theta_{\omega }$ as the orientation of the principal axis with the lower frequency. As a consequence, the estimated ${\hat{\theta }}_{\omega }$, $\theta_{1}$ or $\theta_{1}-\mathrm{sign}\left(\theta_{1}\right)\pi /4$, which ensures p(t) with the lower frequency value will be the actual $\theta_{\omega }$.

Considering the rotated ring-down oscillation is a single tone, almost is monocomponent, along the principal axis, we can utilize the Hilbert transform again to extract the precise frequencies. We construct the analytic signal of p(t) as well as Equation (39)

(52) $$p_{A}\left(t\right)=p\left(t\right)+j\;H\left[p\left(t\right)\right]=E_{p}\left(t\right)\;e^{j\psi_{1}\left(t\right)},$$

where $E_{p}\left(t\right)$ is the instantaneous amplitude of p(t), and $\psi_{1}\left(t\right)$ is the corresponding instantaneous phase, which relates to the instantaneous frequency, $\omega_{1}\left(t\right)$, by

(53) $$\omega_{1}\left(t\right)=\frac{d}{dt}\psi_{1}\left(t\right).$$

**Figure 13:**
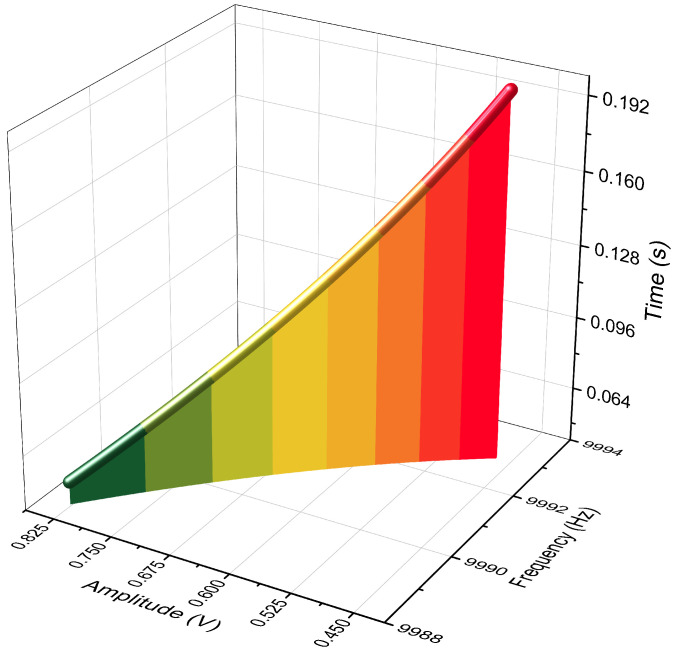
The evolutions of the amplitude and the resonant frequency with time.

Meanwhile, the retrieved instantaneous frequency against the instantaneous amplitude can reveal the nonlinearity in the stiffness. Figure 13 illustrates the evolution of the amplitude and the instantaneous frequency with respect to the time, showing the frequency-amplitude dependency in the ring-down oscillation.

A resonator with the nonlinear stiffness commonly can be described as a duffing oscillator

(54) $$\ddot{p}\left(t\right)+\frac{2}{\tau_{0}}\dot{p}\left(t\right)+\omega_{1}^{2}\left[p\left(t\right)+\sum_{n=2}^{\infty }\gamma_{n}\;p^{n}\left(t\right)\right]=\frac{1}{m}f\left(t\right),$$

where $\gamma_{n}$ is the coefficient of the *n*-th order nonlinearity. Generally, from observation the most dominate nonlinearity is the cubic term in Equation (54), which will lead to the magnification of the force amplitude to the displacement amplitude roughly as

(55) $$M\approx \frac{1/m}{2\sqrt{1/\tau_{0}^{2}+{\left(\omega -\omega_{1}-3\gamma_{3}\omega_{1}E_{P}^{2}/8\right)}^{2}}}.$$

Equation (55) shows the strong dependency between the magnification *M* and the vibration amplitude $E_{p}$. The resonant frequency can be approximated as

(56) $$\omega_{r}\left(t\right)\approx \omega_{1}+\frac{3}{8}\gamma_{3}\;\omega_{1}E_{P}^{2}\left(t\right).$$

Generally, the relation between the amplitude $E_{p}$, in Volts, and the resonant frequency $f_{r}$, in Hz, can be obtained through experiments, then we can fit the relation as

(57) $$f_{r}=aE_{p}^{2}+b,$$

where *a* and *b* are the corresponding coefficients of the fitting. Therefore, the nonlinear coefficient of the cubic stiffness term can be estimated as

(58) $${\hat{\gamma }}_{3}=\frac{8a}{3b}K_{\mathrm{pre}}^{2},$$

where $K_{\mathrm{pre}}$ is the gain of the frond-end circuit which converts the displacement of the resonator to the measured voltage.

## 5. Experiments

**Figure 14:**
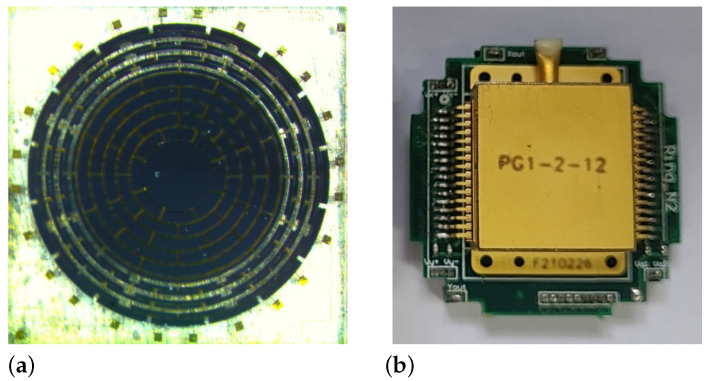
The photos of the resonator (**a**) and the vacuum package (**b**).

The methods discussed in Section 3 and Section 4 were verified by a vacuum-packaged ring resonator shown in Figure 14. The resonator was fabricated by a standard silicon-on-glass processing, which was previously reported in [8].

The chirp forcing voltage for the frequency sweep was generated by a digital-to-analog convertor (DAC) of AD5791 from driven by a Cyclone^®^ IV FPGA of EP4CE from Altera^®^. The readout interface circuit was based on a ring-diode capacitance-to-voltage convertor discussed in [30]. The readout signals were acquired by a dynamic signal analyzer, PXI-4461, from National Instruments.

**Figure 15:**
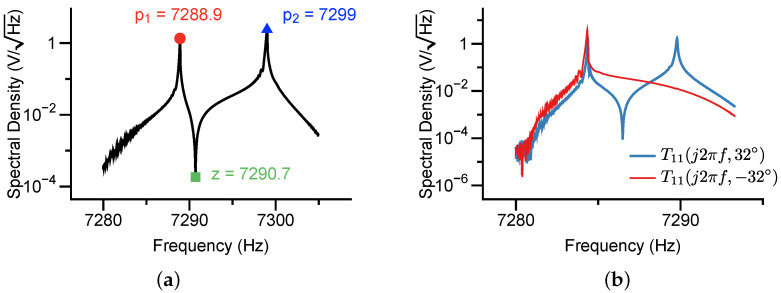
The measured amplitude spectral density of the chirp-driven frequency response from the direction of 0${}^{\circ }$ (**a**). The measured amplitude-frequency responses along the directions of $32^{\circ }$ and $-32^{\circ }$ (**b**). The sweep duration was 70 s and the frequency bandwidth was 30 Hz.

The DAC output a chirp signal with the start frequency of 7280 Hz and the end frequency of 7310 Hz at the duration time of 30 s. The analyzer sampled the signals from the chirp-driven resonator at a rate of 204.8 kHz. The resultant amplitude spectral density is presented in Figure 15a, giving that $p_{1}=7288.9\;\mathrm{Hz}$, z=7290.7Hz, and $p_{2}=7299\;\mathrm{Hz}$. From Equation (26), we can conclude that $|\theta_{\omega }|\approx 32.52^{\circ }$.

To further determine the sign of $\theta_{\omega }$, we additionally excited the resonator along $32^{\circ }$ and $-32^{\circ }$, respectively. The obtained spectrum was demonstrated in Figure 15b, which confirmed that $\theta_{\omega }\approx -32.52^{\circ }$.

**Figure 16:**
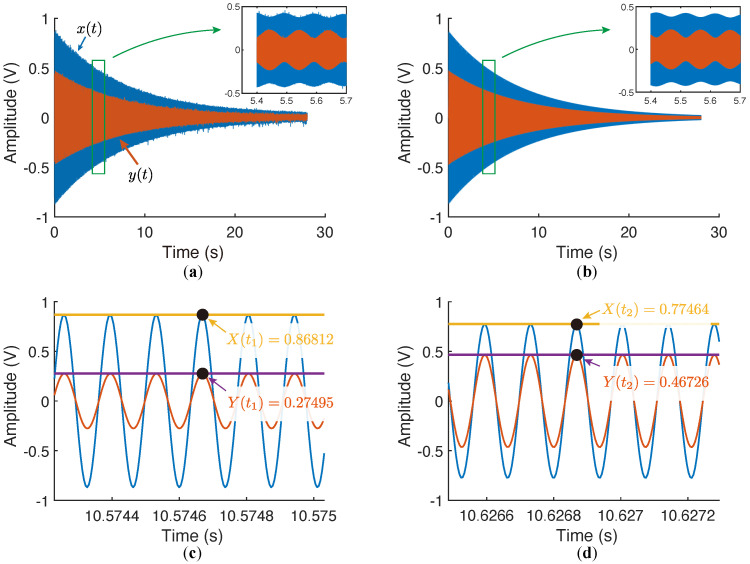
The measured ring-down signals and the peaks and the valleys of the beats. (**a**) The original signals sampled from **XY** directions. (**b**) The signals passed through the bandpass filter. (**c**) Zooming at the position of the peak of X(t). (**d**) Zooming at the position of the peak of Y(t).

The resonator was also characterized by the ring-down method, showing in Figure 16. The resonator was first excited with a phase-locked loop and then released by the loop, yielding a free-oscillation of the working modes. The readout signals from the **xy** directions, x(t) and y(t), were simultaneously sampled and, then, filtered by a narrow bandpass filter to remove the disturbance and noise.

The envelopes of the filtered x(t) and y(t) were obtained via the magnitudes the analytic signals. The time constants of the ring-down decays were given by the exponentially fitted curves of the envelopes: $\tau_{x}=7.652\;s$ and $\tau_{y}=7.662\;s$. By applying Equation (48), we flattened the ring-down signals and attained the peak and the valley values of X(t) and Y(t) as $X(t_{1})=0.86812$, $Y(t_{1})=0.27495$, $Y(t_{2})=0.46726$, and $X(t_{2})=0.77464$, from which $\theta_{\omega }$ would be estimated as $12.17^{\circ }$ or $-32.83^{\circ }$.

**Figure 17:**
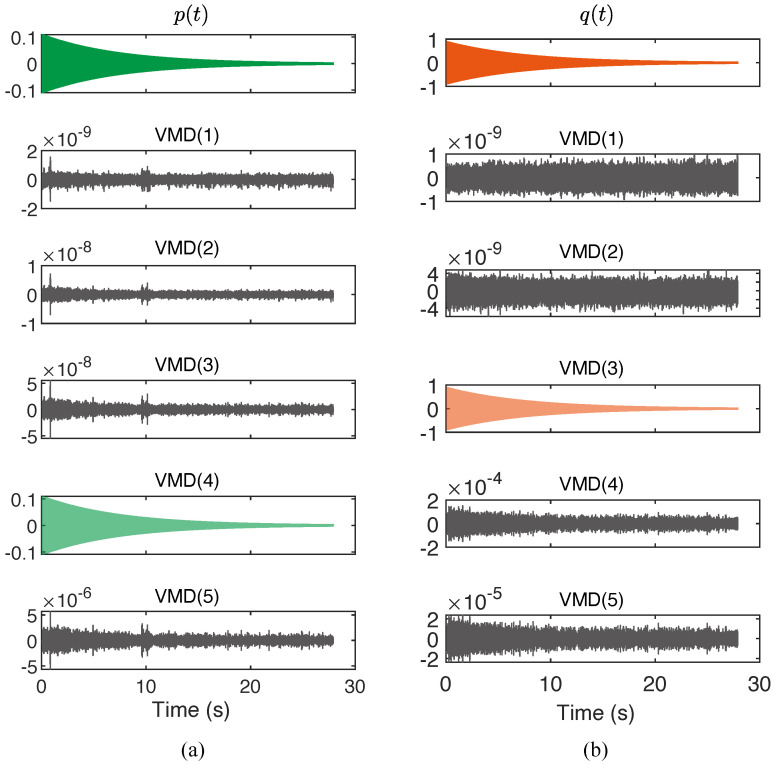
The variational mode decomposition of the modal vibrations (**a**) p(t) and (**b**) q(t).

Once we got the candidates of the modal orientation, we tentatively rotated the oscillations of x(t) and y(t) by $-32.83^{\circ }$, resulting in p(t) and q(t) presented in Figure 17.

To utilize the method described by Equations (52) and (53), the variational mode decomposition (VMD) was respectively performed to p(t) and q(t) to verify the property of monocomponent. It can be readily found that, in both p(t) and q(t), there was only one VMD component whose amplitude significantly greater than the others, suggesting the Hilbert transform can be effectively applied.

**Figure 18:**
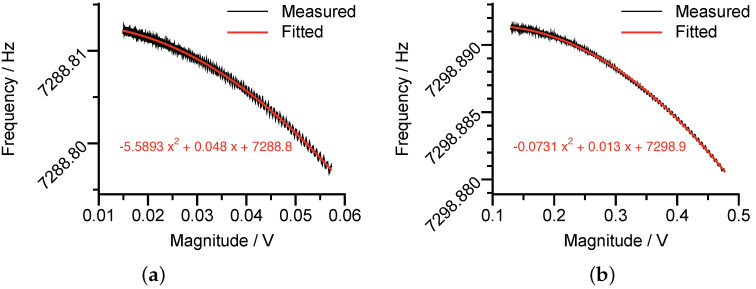
The measured nonlinear stiffness coefficients of the p mode (**a**) and the q mode (**b**).

Finally, the instantaneous frequencies of the ring-down signals were extracted by the numerical differences of the instantaneous phase of the analytic signals constructed by the Hilbert transform, as shown in Figure 18. The attained frequency of p(t) was lower than that of q(t), confirming the modal orientation was $-32.83^{\circ }$. Otherwise, if we rotated x(t) and y(t) by $12.17^{\circ }$ in the first place, the attained frequency order would be opposite, contradicting with the definition.

From Figure 18, the modal frequencies of the working modes were measured as 7288.8 Hz and 7298.9 Hz. In addition, Figure 18 remarkably demonstrated the quadratic relationship between the vibration magnitude and the resonant frequency. From the fitting curves, together with Equation (58), we can calculate the nonlinear coefficients as $\gamma_{31}/K_{\mathrm{pre}}^{2}=2\times 10^{-3}\;V^{-2}$ for **p** mode and $\gamma_{32}/K_{\mathrm{pre}}^{2}=2.67\times 10^{-5}\;V^{-2}$ for **q** mode.

The measured modal frequencies and the orientation by the frequency sweep and the ring-down analysis are summarized in Table 3, where the measured parameters demonstrated good consistence between the two approaches.

## 6. Conclusions

This paper demonstrated the frequency sweep and the ring down approaches to characterize the modal frequencies and the modal orientation of the axisymmetric resonators utilized in the Coriolis vibratory gyroscopes. In the frequency sweep method, the resonator was excited by chirp voltages and the responses were transformed into the frequency domain, within which the zero and the poles imply the modal information of interest. As for resonators with extraordinarily high Q values, it was more convenient to adopt the ring down method, which extracted the modal characterizations, including the modal frequencies, the modal orientation, and the nonlinear stiffness coefficient, by the peaks and the valleys of the beat signals. The results from the experiments were coincident with each other in the two approaches.

## Figures and Tables

**Table 1:** Parameters of the transfer functions in Figure 3.

Parameters	Values	Units
$f_{1}$	9995	Hz
$f_{2}$	10,005	Hz
$f_{0}$	$(f_{1}+f_{2})/2$	Hz
$\tau_{0}$	$10\times 10^{4}/\pi f_{0}$	s
*m*	$1\times 10^{-7}$	kg
$\eta_{k}$	0.04	N/m/V${}^{2}$
$V_{\mathrm{rms}}^{2}$	25.5	V${}^{2}$

**Table 2:** Parameters of the chirp signal shown in Figure 6.

Parameters	Values	Units
$f_{L}$	8000	Hz
$f_{H}$	12,000	Hz
*T*	100	s
*A*	1.5	V

**Table 3:** The measured modal parameters by the frequency sweep and the ring-down analysis.

Parameters	Frequency Sweep	Ring-Down	Units
$\omega_{1}$	7288.9×2π	7288.8×2π	rad/s
$\omega_{2}$	7299.0×2π	7298.9×2π	rad/s
$\theta_{\omega }$	−32.52	−32.83	${}^{\circ }$
$\gamma_{31}/K_{\mathrm{pre}}^{2}$	–	$2\times 10^{-3}$	$V^{-2}$
$\gamma_{32}/K_{\mathrm{pre}}^{2}$	–	$2.67\times 10^{-5}$	$V^{-2}$
